# Exploring the mechanism of ShenGui capsule in treating heart failure based on network pharmacology and molecular docking: A review

**DOI:** 10.1097/MD.0000000000037512

**Published:** 2024-04-05

**Authors:** Xiang Luo, Yunke Shi, Yiming Ma, Yixi Liu, Pan Jing, Xingyu Cao, Jincheng Wang, Zhao Hu, Hongyan Cai

**Affiliations:** a Department of Cardiology, the First Affiliated Hospital of Kunming Medical University, Kunming, China.

**Keywords:** heart failure, IL-1β, molecular docking, network pharmacology, SGC

## Abstract

ShenGui capsule (SGC), as a herbal compound, has significant effects on the treatment of heart failure (HF), but its mechanism of action is unclear. In this study, we aimed to explore the potential pharmacological targets and mechanisms of SGC in the treatment of HF using network pharmacology and molecular docking approaches. Potential active ingredients of SGC were obtained from the traditional Chinese medicine systems pharmacology database and analysis platform database and screened by pharmacokinetic parameters. Target genes of HF were identified by comparing the toxicogenomics database, GeneCards, and DisGeNET databases. Protein interaction networks and gene-disorder-target networks were constructed using Cytoscape for visual analysis. Gene ontology and Kyoto Encyclopedia of Genes and Genomes were also performed to identify protein functional annotations and potential target signaling pathways through the DAVID database. CB-DOCK was used for molecular docking to explore the role of IL-1β with SGC compounds. Sixteen active ingredients in SGC were screened from the traditional Chinese medicine systems pharmacology database and analysis platform, of which 36 target genes intersected with HF target genes. Protein-protein interactions suggested that each target gene was closely related, and interleukin-1β (IL-1β) was identified as Hub gene. The network pharmacology analysis suggested that these active ingredients were well correlated with HF. Kyoto Encyclopedia of Genes and Genomes enrichment analysis suggested that target genes were highly enriched in pathways such as inflammation. Molecular docking results showed that IL-1β binds tightly to SGC active components. This experiment provides an important research basis for the mechanism of action of SGC in the treatment of HF. In this study, the active compounds of SGC were found to bind IL-1β for the treatment of heart failure.

## 1. Introduction

Heart failure (HF) is a major global health problem, the endpoint of almost all cardiovascular diseases, characterized by high morbidity and mortality, and also poses a serious economic burden. For US statistics alone, the prevalence of HF is projected to increase by 46% from 2012 to 2030 and increase with population growth and aging; the proportion of the total population with HF is projected to increase from 2.4% in 2012 to 3.0% in 2030.^[1–3]^ The pathological mechanisms of HF are intricate and complex, including abnormal calcium cycling, activation of the neurohumoral system, myocardial cell damage, inflammatory response, and ventricular remodeling.^[4]^ It has been reported that inflammation is a cause and consequence of HF and plays an important role in the mechanism and progression of HF,^[5]^ and that some inflammatory markers can be used as independent predictors of HF.^[6]^ Inflammatory factors such as interleukin-1β (IL-1β) and tumor necrosis factor (TNF) are highly expressed in the heart of HF patients compared to healthy individuals, and the activation of inflammation induces left ventricular remodeling and exacerbates cardiac dysfunction.^[7–9]^ Experimental studies have shown that high inflammatory expression is associated with the activation of the renin-angiotensin-aldosterone system and immune response mechanisms, and correlates with disease severity and prognosis.^[8–10]^ The “golden quadruple” (angiotensin receptor neprilysin inhibitor/angiotensin-converting enzyme inhibitor [ARNI/ACEI], mineralocorticoid receptor antagonist, β-blocker, sodium-glucose co-transporter 2 inhibitor) is currently the basic treatment for HF,^[2]^ which can effectively reduce inflammation and improve cardiac dysfunction.^[11–14]^ However, there are individual differences in the efficacy of all medications, as well as contraindications and complications with prolonged use of medications, such as hypotension, angioedema, dry cough, and urinary tract infections.^[2,15,16]^ Therefore, we need to explore clinical drugs that are multi-targeted, effective, have few side effects, and can be used as complementary and alternative drugs for primary and secondary prevention of HF.^[17]^

Chinese herbal medicines contain many active ingredients that can act through a variety of pathways, including antiinflammatory, antioxidative stress, metabolic modulation, antifibrotic, and pro-angiogenic mechanisms in the treatment of HF.^[18]^ ShenGui capsule (SGC), as a traditional Chinese herbal compound, contains 3 components: Red Ginseng, Chuanxiong, and Gui Zhi. Studies have confirmed that SGC can effectively treat cardiovascular diseases,^[19]^ SGC has efficient effects in antioxidative stress, endothelial cell protection, antiapoptosis, and inhibition of proinflammatory cytokine and chemokine expression.^[20–22]^ Wang et al^[19]^ confirmed that SGC can improve cardiac function, which was shown by plasma metabolomics to be associated with regulating energy metabolism, and inflammatory response. Some clinical studies in small samples also reported that SGC can reduce inflammation levels and improve cardiac function in patients with chronic HF, but further explanation at the molecular level is lacking.

**Figure 1.**
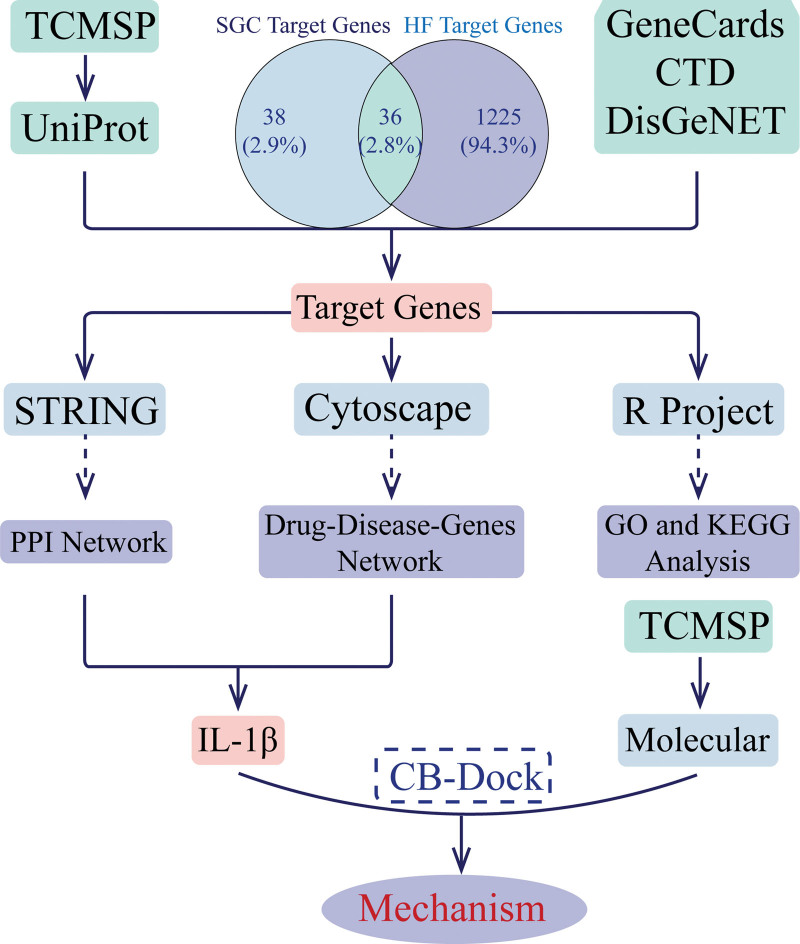
The flowchart of this study. CTD = comparing the toxicogenomics database, GO = gene ontology, HF = heart failure, KEGG = Kyoto Encyclopedia of Genes and Genomes, PPI = protein-protein interaction, SGC = ShenGui capsule, TCMSP = the traditional Chinese medicine systems pharmacology database and analysis platform.

From the perspective of disease modules, network pharmacology suggests that drugs can simultaneously target multiple proteins in the signaling module of a disease, moving away from the current therapeutic paradigm of “one disease—one target—one drug” and emphasizing holistic, systemic, and drug-drug interactions.^[23,24]^ Molecular docking is a well-established computer-based structural approach that predicts the biological activity of drugs by evaluating the interactions between compounds and potential targets.^[25,26]^ Network pharmacology^[24]^ and molecular docking techniques^[26]^ have systematically revealed the multiple effects of drug, gene, and disease relationships. In this paper, the active ingredients of SGC and HF were first screened to obtain effective target genes. Then the target genes and target pathways were investigated by network pharmacology, gene ontology (GO), and biological pathway (Kyoto Encyclopedia of Genes and Genomes [KEGG]) function enrichment, and molecular docking techniques using target genes. This study is the first to reveal the molecular mechanisms, signaling pathways, and molecular targets of SGC for the treatment of HF based on network pharmacology and molecular docking techniques, and to provide a scientific basis for the use of SGC in the treatment of HF (Fig. 1 shows the flowchart of this study).

## 2. Materials and methods

### 2.1. Active ingredients and target genes of SGC

According to reports, there are currently traditional Chinese medicine (TCM) databases related to the TCM systems pharmacology database and analysis platform (TCMSP), Chem-TCM, Herbal Ingredients' Targets Platform 2.0, etc. However, different databases have different focuses and lack timely updates. Then, we have chosen TCMSP (https://tcmsp-e.com/tcmsp.php), which is unique but has comprehensive data and functions, as our drug database.^[27]^ The main active compounds were searched in the TCMSP database. Active ingredients and target genes were obtained by screening based on absorption, distribution, metabolism and excretion properties of drugs with oral bioavailability ≥30% and drug similarity ≥0.18. Target genes were corrected by Uniport (https://www.uniprot.org/) protein database.

### 2.2. Target genes for HF

To improve the reliability of disease genes, we selected 3 commonly used and highly reliable disease databases to obtain our final target genes by intersecting them. The target genes of HF were identified by comparing the toxicogenomics database (http://ctdbase.org/), GeneCards (https://www.genecards.org/), and DisGeNET (https://www.disgenet.org/) databases.

### 2.3. Network construction

The drug-disease target genes were obtained by taking the intersection of drug target genes and disease target genes, and the protein-protein interaction (PPI) network analysis of the target genes was constructed by STRING (https://cn.string-db.org/) database, and the Hub genes were obtained according to the MCC algorithm in CytoHubba. Then the drug-disease gene network was constructed by Cytoscape 3.9.0, and the relationship between compounds, target genes, and diseases was analyzed.

### 2.4. GO and KEGG enrichment analysis

GO and KEGG pathway enrichment analyses of target genes were performed based on the DAVID (https://david.ncifcrf.gov/) online database and visualized by R version 4.1.2.

### 2.5. Molecular docking of Hub gene to active ingredients

SGC compound structures and protein crystal structures were obtained from PubChem (https://pubchem.ncbi.nlm.nih.gov/) and RCSB (http://www.rcsb.org/) databases, respectively. Molecular docking was then performed using CB-Dock (http://cao.labshare.cn/cb-dock//) to explore the docking of selected active components of the target gene network to the IL-1β receptor. The lower the Vina score, the more stable the binding of the ligand to the receptor, and was used to initially assess the binding activity of the compounds to the target.

## 3. Results

### 3.1. Active ingredients and target genes of SGC

According to the introduction of SGC, 3 ingredients were identified: ChuanXiong, HongShen, GuiZhi; the active ingredients of the 3 ingredients were obtained separately, and a total of 16 compounds were obtained by screening oral bioavailability ≥30% and drug similarity ≥0.18 based on pharmacokinetics through the TCMSP database (Table 1). The predicted target genes were screened by the compound names, and then the gene names were converted and duplicate corrections were removed by Uniport database; 74 target genes were eventually obtained by screening 13 effective compounds as 3 compounds failed to predict the target genes (Table 2).

**Table 1:** Effective compounds of SGC.

Mol ID	Molecule name	OB (%)	DL
MOL002032	DNOP	40.59	0.4
MOL002372	(6Z, 10E, 14E, 18E)-2,6,10,15,19,23-hexamethyltetracosa-2,6,10,14,18,22-hexaene	33.55	0.42
MOL000358	β-sitosterol	36.91	0.75
MOL005344	Ginsenoside rh2	36.32	0.56
MOL001736	(−)-Taxifolin	60.51	0.27
MOL000359	Sitosterol	36.91	0.75
MOL000492	(+)-Catechin	54.83	0.24
MOL000073	ent-Epicatechin	48.96	0.24
MOL004576	Taxifolin	57.84	0.27
MOL011169	Peroxyergosterol	44.39	0.82
MOL001494	Mandenol	42	0.19
MOL002135	Myricanone	40.6	0.51
MOL002140	Perlolyrine	65.95	0.27
MOL002151	Senkyunone	47.66	0.24
MOL002157	Wallichilide	42.31	0.71
MOL000433	FA	68.96	0.71

DL = drug similarity, OB = oral bioavailability, SGC = ShenGui capsule.

**Table 2:** Target genes of effective compounds of SGC.

Mol ID	Target genes
MOL002032	SCN5A, ADRB2, CHRM3, CHRM1, ADRA1B
MOL000358	PGR, NCOA2, PTGS1, PTGS2, HSP90, PIC3KG, KCNH2, DRD1, CHRM3, CHRM1, SCN5A, GABRA2, CHRM4, PDE3A, HTR2A, GABRA5, ADRA1A, GABRA3, PON1, CHRM2, ADRA1B, ADRB2, CHRNA2, SLC6A4, OPRM1, GABRA1, CHRNA7, BCL2, BAX, CASP9, JUN, CASP3, CASP8, PRKCA, TGFB1, MAP2
MOL005344	BAX, TNF, CASP3, PTGS2, NFKBIA, IL1B, CASP1, ADCYAP1, PSMG1, MAP2K4, SLC2A4, IFNG
MOL001736	PTGS1, PTGS2, HSP90, PIC3KG
MOL000359	PGR, NCOA2, NR3C2
MOL000492	PTGS1, ESR1, PTGS2, HSP90, NCOA2, CALM, RXRA, CAT, HAS2
MOL000073	PTGS1, ESR1, PTGS2, HSP90
MOL004576	PTGS1, PTGS2, HSP90, PIK3CG, RXRA, AR, RELA, MAC1, ICAM1, DGAT2, MTTP, APOB
MOL001494	PTGS1, PTGS2, NCOA2
MOL002135	NOS2, PTGS1, KCNH2, ESR1, SCN5A, PPARG, PTGS2, F7, RXRA, PDE3A, ADRB2, ESR2, DPP4, MAPK14, GSK3B, HSP90, CDC2, CHEK1, IGHG1, Pim-1, CCNA2, NCOA1, VEGFR2, AR
MOL002140	PTGS2, RXRA
MOL002157	PTGS2, NR3C2, NR3C1, NCOA2
MOL000433	CDC2, GSK3B

ADCYAP1 = pituitary adenylate cyclase-activating polypeptide, ADRA1A = α-1A adrenergic receptor, ADRA1B = α-1B adrenergic receptor, ADRB2 = β-2 adrenergic receptor, APOB = apolipoprotein B-100, AR = aldose reductase, BAX = apoptosis regulator BAX, BCL-2 = apoptosis regulator Bcl-2, CALM = calmodulin, CASP1 = caspase-1, CASP3 = caspase-3, CASP8 = caspase-8, CASP9 = caspase-9, CAT = catalase, CCNA2 = cyclin-A2, CDC2 = cell division protein kinase 2, CHEK1 = serine/threonine-protein kinase Chk1, CHRM1 = muscarinic acetylcholine receptor M1, CHRM2 = muscarinic acetylcholine receptor M2, CHRM3 = muscarinic acetylcholine receptor M3, CHRM4 = muscarinic acetylcholine receptor M4, CHRNA2 = neuronal acetylcholine receptor subunit α-2, CHRNA7 = neuronal acetylcholine receptor protein, α-7 chain, DGAT2 = diacylglycerol O-acyltransferase 2, DPP4 = dipeptidyl peptidase IV, DRD1 = dopamine D1 receptor, ESR1 = estrogen receptor, ESR2 = estrogen receptor β, F7 = coagulation factor VII, GABRA1 = γ-aminobutyric acid receptor subunit α-1, GABRA2 = γ-aminobutyric-acid receptor α-2 subunit, GABRA3 = γ-aminobutyric-acid receptor α-3 subunit, GABRA5 = γ-aminobutyric-acid receptor α-5 subunit, GSK3B = glycogen synthase kinase-3 β, HAS2 = hyaluronan synthase 2, HSP90 = heat shock protein HSP 90, HTR2A = 5-hydroxytryptamine 2A receptor, ICAM1 = intercellular adhesion molecule 1, IFNG = interferon γ, IGHG1 = Ig γ-1 chain C region, IL1B = interleukin-1 β, JUN = transcription factor AP-1, KCNH2 = potassium voltage-gated channel subfamily H member 2, MAC1 = metal-binding activator 1, MAP2 = microtubule-associated protein 2, MAP2K4 = dual specificity mitogen-activated protein kinase 4, MAPK14 = mitogen-activated protein kinase 14, MTTP = microsomal triglyceride transfer protein large subunit, NCOA1 = nuclear receptor coactivator 1, NCOA2 = nuclear receptor coactivator 2, NFKBIA = NF-κ-B inhibitor α, NOS2 = nitric oxide synthase, inducible, NR3C1 = glucocorticoid receptor, NR3C2 = mineralocorticoid receptor, OPRM1 = µ-type opioid receptor, PDE3A = CGMP-inhibited 3’,5’-cyclic phosphodiesterase A, PGR = progesterone receptor, PIC3KG = phosphatidylinositol-4,5-bisphosphate 3-kinase catalytic subunit, gamma isoform, PIK3CG = phosphatidylinositol-4,5-bisphosphate 3-kinase catalytic subunit, gamma isoform, Pim-1 = proto-oncogene serine/threonine-protein kinase Pim-1, PON1 = serum paraoxonase/arylesterase 1, PPARG = peroxisome proliferator activated receptor gamma, PRKCA = protein kinase C α type, PSMG1 = proteasome assembly chaperone 1, PTGS1 = prostaglandin G/H synthase 1, PTGS2 = prostaglandin G/H synthase 2, RELA = transcription factor p65, RXRA = retinoic acid receptor RXR-α, SCN5A = sodium channel protein type 5 subunit α, SLC2A4 = solute carrier family 2, facilitated glucose transporter member 4, SLC6A4 = sodium-dependent serotonin transporter, TGFB1 = transforming growth factor β-1, TNF = tumor necrosis factor, VEGFR2 = vascular endothelial growth factor receptor 2.

### 3.2. Target genes of HF

**Figure 2.**
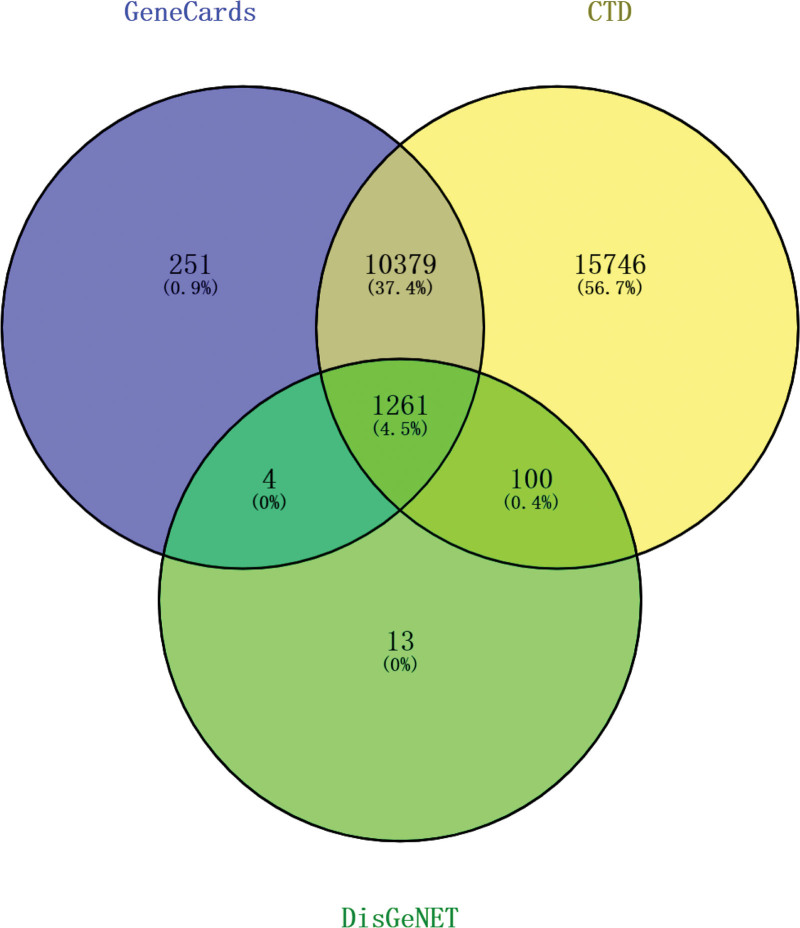
Target genes for heart failure. CTD= = comparing the toxicogenomics database.

To improve the confidence of target genes, 1261 common target genes for HF were obtained by jointly comparing the toxicogenomics database, GeneCards, and DisGeNET databases with the online tool Venny 2.1.0 (Fig. 2).

### 3.3. Drug-disease common target genes

**Figure 3.**
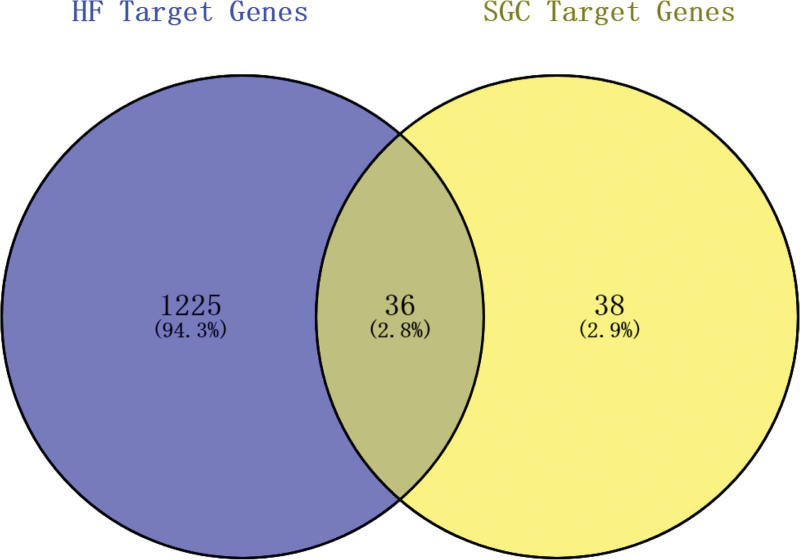
Common target genes of HF and SGC. HF = heart failure, SGC = ShenGui capsule.

The intersection of drug target genes and disease target genes was constructed by the online web tool Venny 2.1.0 with 36 target genes in common (Fig. 3).

### 3.4. Network construction and analysis

#### 3.4.1. Construction and analysis of protein interaction network

**Figure 4.**
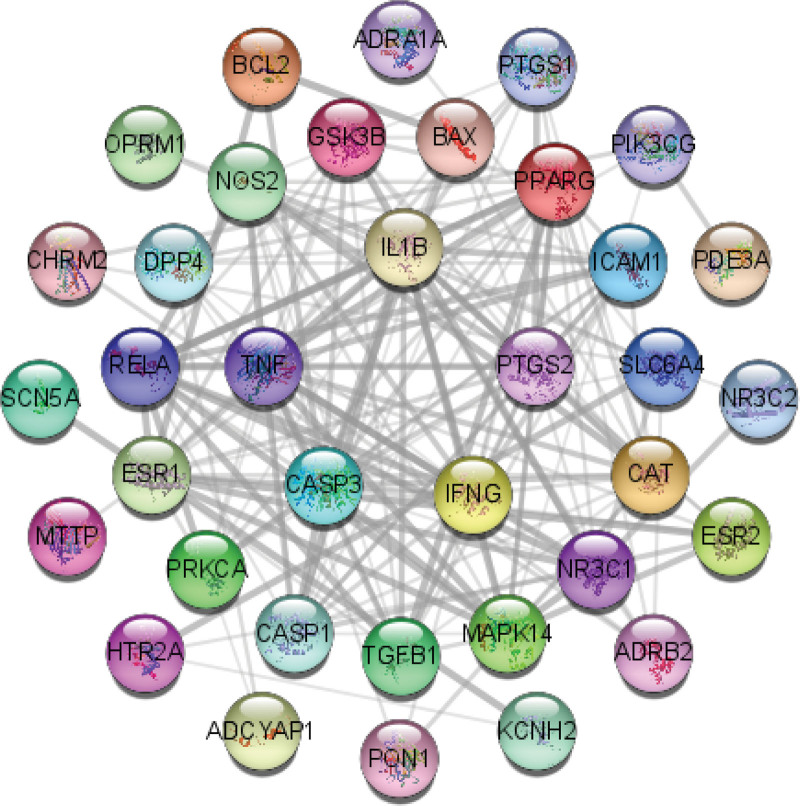
PPI network (the nodes indicate 36 genes, and the black connecting lines represent the interactions of genes). PPI = protein-protein interaction.

**Figure 5.**
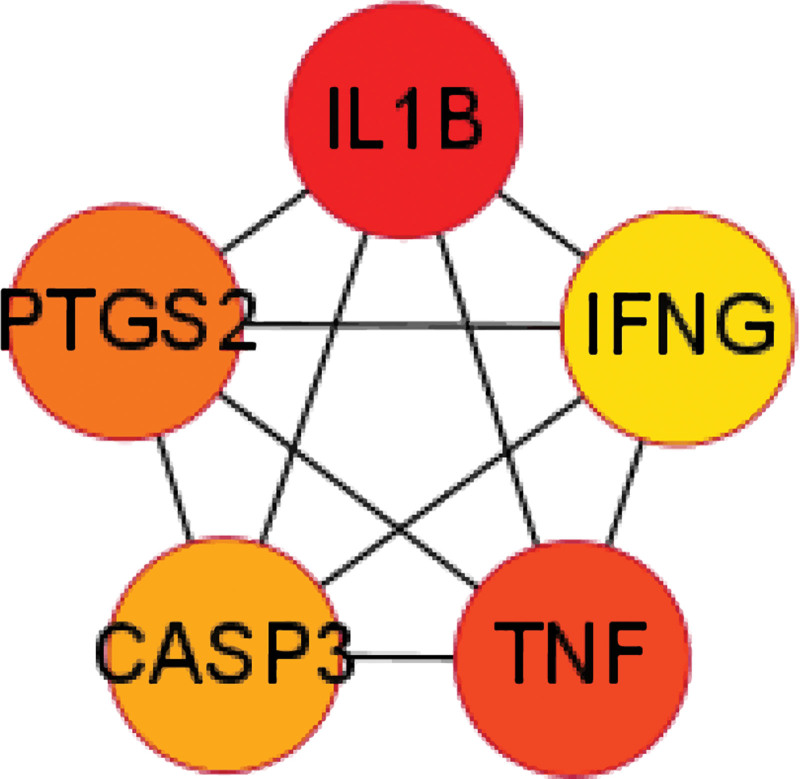
Top 5 genes (the top 5 genes obtained according to the MCC algorithm in the PPI network). PPI = protein-protein interaction, TNF = tumor necrosis factor.

The PPI network map was constructed by the STRING database, and visualized and analyzed by Cytoscape 3.9.0 (Fig. 4), including 36 nodes and 171 edges, and the Hub gene screening was performed by the MCC algorithm in Cytohubba, and the top five genes in order were IL1B, TNF, PTGS2, CASP3, and IFNG (Fig. 5).

#### 3.4.2. Disease-drug-target genes network diagram

**Figure 6.**
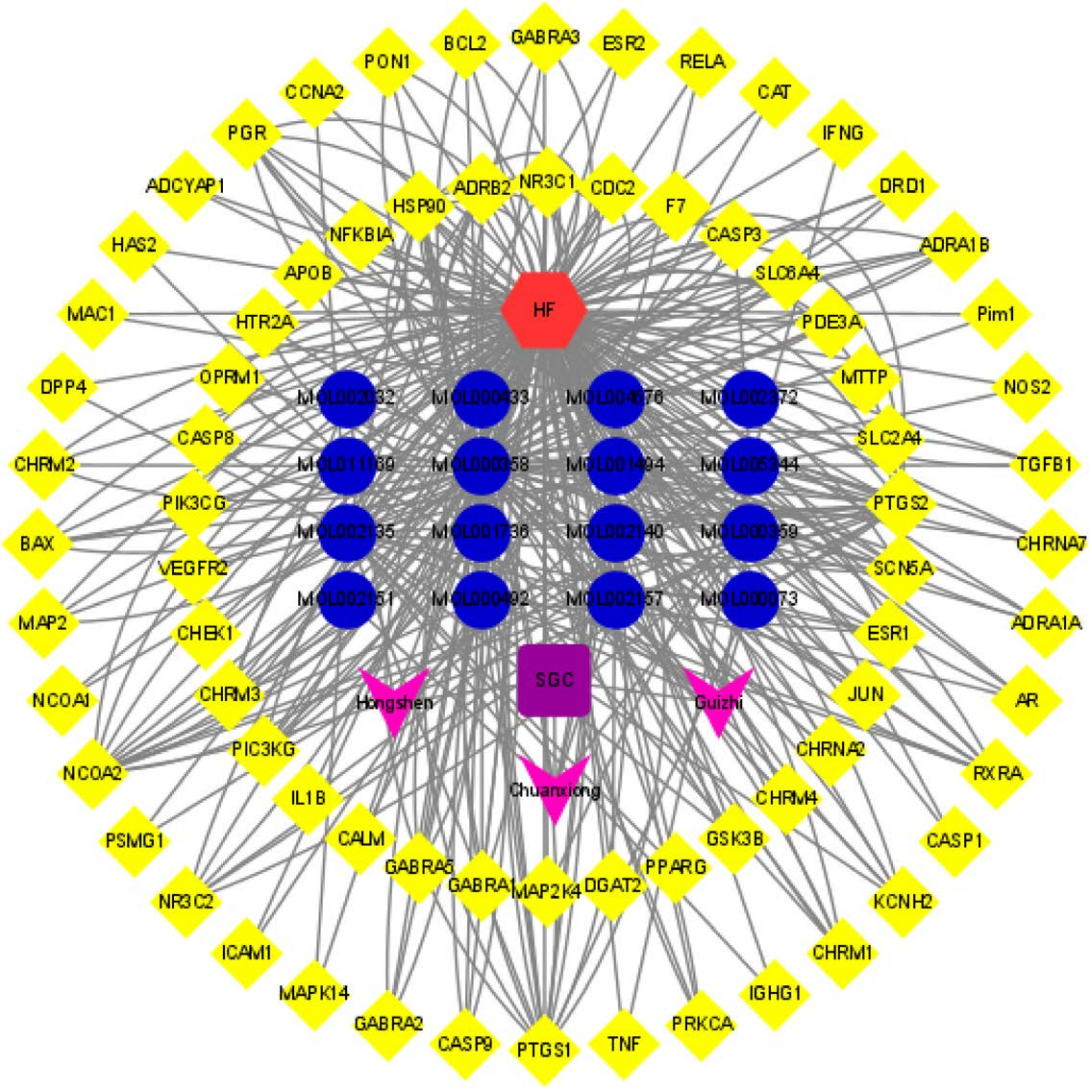
Disease-drug-compound-target genes network (red hexagon represents the disease HF, purple square represents the drug SGC, blue dot represents the active compounds, pink V represents the components of SGC; yellow diamond is the name of target genes). HF = heart failure, SGC = ShenGui capsule.

To further visualize the association between diseases and drugs, a disease-drug-compound-target gene network map was constructed using Cytoscape 3.9.0 (Fig. 6).

### 3.5. GO and KEGG enrichment analysis

**Figure 7.**
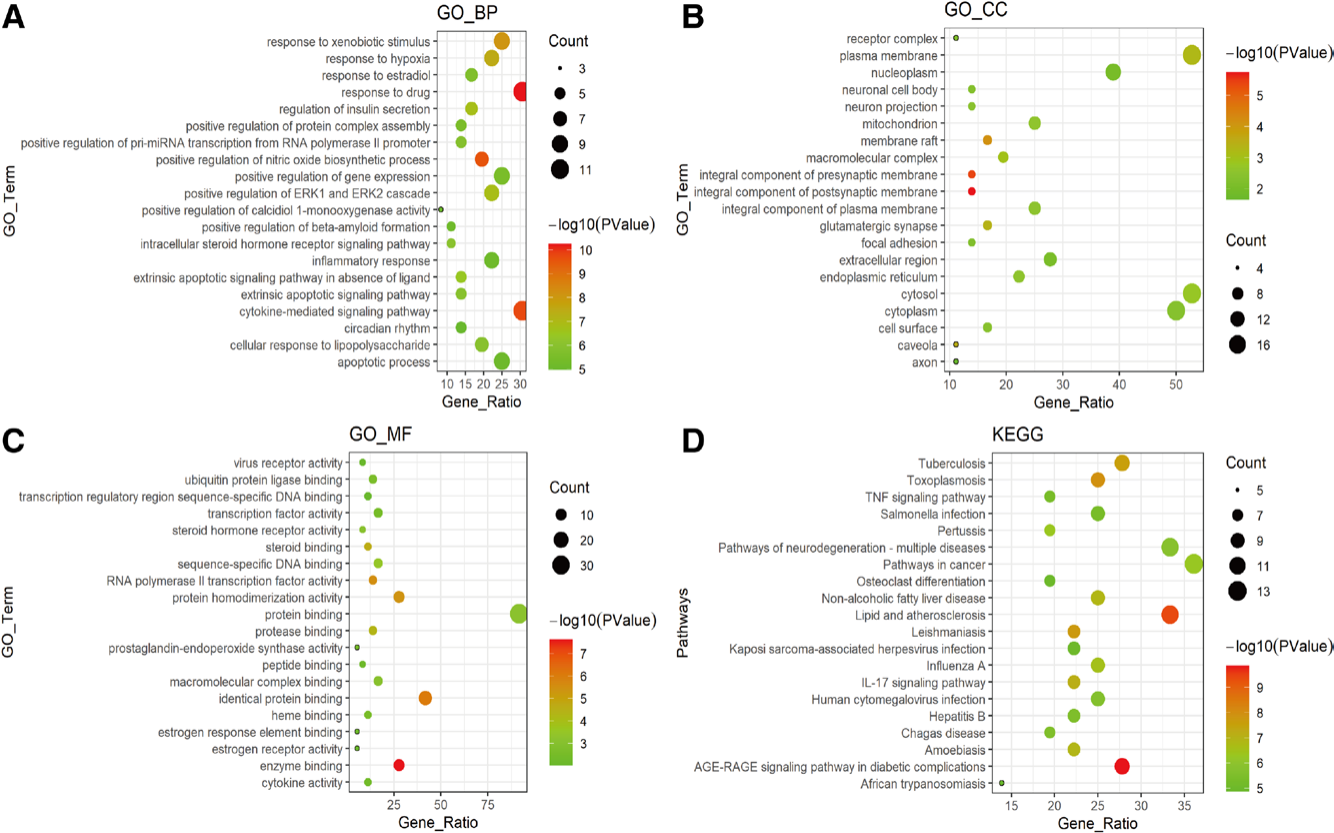
Top 20 results of GO and KEGG pathway enrichment analysis of target genes. (A) Enriched BP of target genes. (B) Enriched CC of target genes. (C) Enriched MF of target genes. (D) Enriched KEGG pathways of target genes. CC = cellular component, GO = gene ontology, KEGG = Kyoto Encyclopedia of Genes and Genomes, MF = molecular function.

Based on the online DAVID database, biological function (GO) and pathway enrichment (KEGG) analysis of drug-disease target genes were performed (*P* < .05) to further explain the mechanism of action of SGC for HF treatment in terms of protein function annotation and potential target signaling pathways; some results were visualized by R 4.1.2 ggplot2 package. Biological processes were mainly enriched in response to drug, response to hypoxia, inflammatory responses, etc (Fig. 7A); cellular components were enriched in macromolecular complex, plasma membrane, cell surface, etc (Fig. 7B); molecular functions are enriched in enzyme binding, protease binding, steroid binding, etc (Fig. 7C); the pathway enrichment (KEGG) analysis was mainly enriched in TNF signaling, NF-kB signaling, apoptosis, and other signaling pathways (Fig. 7D).

### 3.6. Molecular docking of IL-1β

**Figure 8.**
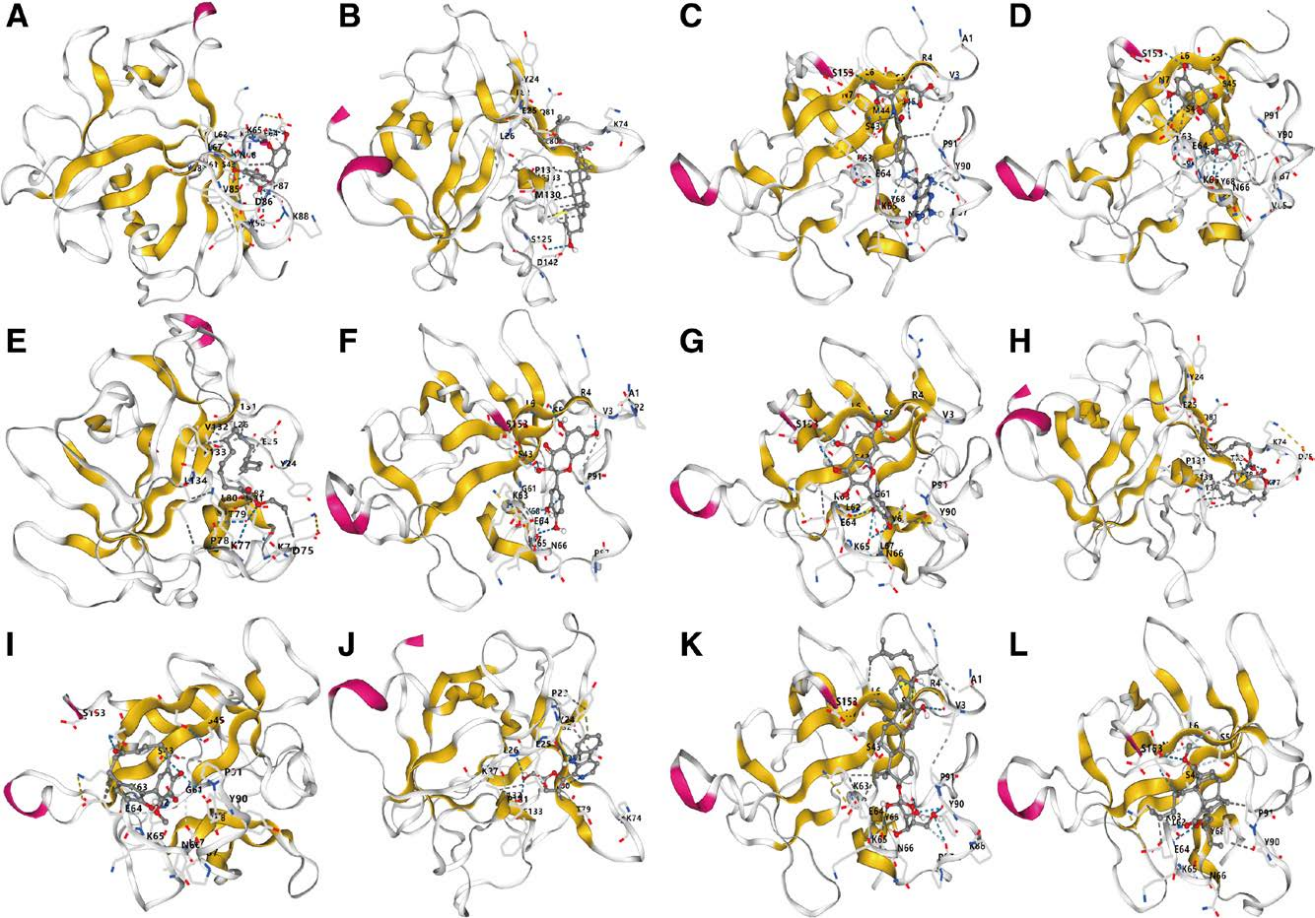
Molecular docking of the receptors and their ligands. (A) ent-Epicatechin to IL-1β. (B) β-sitosterol/sitosterol to IL-1β. (C) FA to IL-1β. (D) (+)-Catechin to IL-1β. (E) Mandenol to IL-1β. (F) (−)-Taxifolin to IL-1β. (G) Taxifolin to IL-1β. (H) DNOP to IL-1β. (I) Myricanone to IL-1β. (J) Perlolyrine to IL-1β. (K) Ginsenoside rh2 to IL-1β. (L) Wallichilide to IL-1β.

To further reveal the mechanism of action between compounds and target genes, the structures of target gene IL-1β (PDB: 1T4Q) and some compounds were separately obtained from RCSB and PubChem databases, and then molecular docking was performed by the online tool CB-DOCK to understand the corresponding binding sites (Fig. 8). The lower Vina scores indicate a stronger and more stable interaction between the compound and the receptor (Table 3).

**Table 3:** Compounds and IL-1β molecular docking table.

Mol ID	Molecule name	Vina	Cavity	Center			Size		
		Score	Size	*x*	*y*	*z*	*x*	*y*	*z*
MOL000073	ent-Epicatechin	−6.7	158	−16	9	−14	20	20	20
MOL000358 MOL000359	β-sitosterol Sitosterol	−7.7	111	−19	4	5	25	25	25
MOL000433	FA	−7.2	158	−16	9	−14	28	28	28
MOL000492	(+)-Catechin	−6.6	158	−16	9	−14	21	21	21
MOL001494	Mandenol	−4.7	111	−19	4	5	26	26	26
MOL001736	(−)-Taxifolin	−6.8	158	−16	9	−14	21	21	21
MOL004576	Taxifolin	−7.1	158	−16	9	−14	21	21	21
MOL002032	DNOP	−4.5	111	−19	4	5	26	26	26
MOL002135	Myricanone	−6.9	158	−16	9	−14	20	20	20
MOL002140	Perlolyrine	−6.7	111	−19	4	5	21	21	21
MOL005344	Ginsenoside rh2	−7.5	158	−16	9	−14	28	28	28
MOL002157	Wallichilide	−6	158	−16	9	−14	21	21	21

## 4. Discussion

HF, as a chronic and complex clinical syndrome, has increased in incidence and although the overall hospitalization and mortality rates have decreased slightly, it is still at a high value and still poses a serious threat to human life, health, and economy,^[1,28]^ and we still need to explore effective treatments.SGC is an effective drug for the treatment of HF,^[19]^ but the molecular mechanism of action is unclear. This study reveals for the first time that SGC for HF may alleviate cardiac dysfunction by regulating the expression of IL-1β and inhibiting the activation of inflammation through network pharmacology and molecular docking techniques.

Studies have shown that the main components of SGC can treat cardiovascular diseases through multiple mechanisms of action and multiple targets.^[29,30]^ SGC has a definite efficacy in the treatment of HF, and the apparent mechanism of action may be anti-inflammatory. Sixteen active compounds extracted from SGC have been extensively studied and shown to have significant effects on inflammation. For example, β-sitosterol can inhibit the expression of pro-inflammatory factors IL-1β, IL-6, IL-12, etc, controlling the inflammatory response in rheumatoid arthritis in mice^[31]^; ginsenoside rh2 acts on TLR4/PI3K/Akt/mTOR, Raf-1/MEK/ ERK, and Keap1/Nrf2/HO-1 signaling pathways to reduce lipopolysaccharide-induced pulmonary edema and neutrophil infiltration, inhibit the expression of inflammatory factors TNF-α, IL-1β, IL-4, and IL-6, suppress oxidative stress, and reduce lung injury^[32,33]^; taxifolin inhibits inflammatory mediators and reduces lung injury through NF-kB, MAPKs, and AKT signaling pathways in a variety of diseases.^[34,35]^ (−)-Epicatechin can treat cardiovascular diseases, cancer, and diabetes through antioxidative stress, antiinflammation, interference with mitochondrial metabolism, and inhibition of cell signaling.^[36,37]^ In conclusion, these active ingredient compounds act on a variety of cytokines and signaling pathways with varying degrees of therapeutic effects on HF.

In the disease-drug-compound-target genes network, we found that multiple compounds of SGC predicted multiple target genes, including TNF, IL-1β, PTSG2, CASP3, PPARG, NCOA2, etc, confirming that herbal therapy works through multiple targets and pathways. The PPI network analysis suggested that the genes were interlinked and acted together. The top 5 genes obtained by the MCC algorithm in Cytohubba were all immune inflammation-related genes (IL1B, TNF, PTGS2, CASP3, IFNG), which is consistent with previous studies that inflammation is an important pathogenesis of HF, and the mechanism of SGC for HF treatment is focused on the regulation of immune inflammation. The cytokine hypothesis^[38]^ suggests that HF progresses due to a cascade of cytokine responses that disrupt the relative balance between cardiac pathways of pathological inflammation and tissue repair processes (physiological inflammation),^[38,39]^ and that high expression of inflammatory factors regulates protein synthesis disrupting cytoplasmic calcium processing, leading to negative inotropy, which affects reduced LV ejection capacity and poor ventricular remodeling, exacerbating cardiac dysfunction.^[40,41]^ In both clinical and basic studies, it has been demonstrated that high expression of inflammatory factors such as IL-1β, TNF-α, IL-6, and NLRP3 inflammatory vesicles in the myocardium leads to progressive cardiac dilatation and HF.^[42,43]^ Between the important role of inflammation in the mechanism and progression of HF, antiinflammatory therapy such as inhibition of TNF-α, IL-1β, and IL-6 would be a potential biological target for the treatment of HF.^[5]^ Evidence suggests that Enbrel targeting TNF-α for advanced HF is safe and well tolerated, improving cardiac function and left ventricular remodeling,^[44,45]^ but subsequent multicenter studies RENEWAL (RENAISSANCE and RECOVER) and ATTACH trials have shown that anti-TNF-α therapy does not have a beneficial effect on HF mortality and hospitalization rates, but rather increased the risk of infection.^[46,47]^ However, in basic and clinical trials it was shown that exercise and IL-1β antibody treatment reduced inflammation in HF and improved LV remodeling or dysfunction and coronary artery dysfunction to reduce HF.^[48,49]^ A subsequent randomized, double-blind trial including 10,061 samples showed that treatment with the IL-1β inhibitor canakinumab reduced hospitalization and all-cause mortality in HF.^[50]^ Based on the PPI network suggesting that IL-1β is the most highly associated inflammatory factor, and that antagonizing IL-1β is effective in treating HF, we consider that SGC may exert its effect by regulating IL-1β.

GO and KEGG enrichment analysis of 36 common target genes through the DAVID database showed that a total of 286 biological functions and 93 signaling pathways were annotated, and according to the enrichment results of KEGG pathways, inflammatory signaling pathways such as TNF signaling, IL-17 signaling, NF-kB signaling, TOLL-like receptors, and NOD-like receptors were enriched, suggesting that these bioinformatics functions and inflammatory signaling pathways are important mechanisms of action of SGC in the treatment of HF. Studies have shown that NLRP3, Toll-like receptors, and NF-kB signaling pathways play their important roles in the pathogenesis of HF,^[51,52]^ and that inhibition of inflammation and inflammatory pathways delays the development of HF. In a rat model of HF, it was demonstrated that inflammatory factors, NF-kB P65, and TLR were abundantly expressed in the failing heart, and inhibition of TLR and NF-kB attenuated the expression of inflammatory factors such as IL-1β in the heart, LV remodeling, and improved LV dilation and cardiac dysfunction in rats.^[53,54]^ Ye et al,^[55]^ in patients with chronic HF, found that plasma exosomal mtDNA of IL-1β and IL-8 expression and secretion were increased, exacerbating the inflammatory response in HF through the TLR9-NF-kB pathway; Foldes et al,^[56]^ found a significant increase in TLR4 expression in monocytes from patients with HF, and inhibition of the Toll-like receptor signaling pathway in monocytes resulted in relief of clinical symptoms of HF and improvement of cardiac function in patients.

According to the results of molecular docking, all the active compound components were able to bind stably to IL-1β, while sitosterol and β-sitosterol were the best-binding molecules. Previous studies have shown that sitosterol and β-sitosterol inhibited the activation of TLR4 and NF-kB pathways and reduced the expression of inflammatory factors such as IL-1β, TNF-α, and IL-6.^[57,58]^ Despite our study reveals that the therapeutic mechanism of SGC for HF may be to reduce the overexpression of inflammation by modulating the expression of cytokines such as IL-1β, thereby improving the dysfunction of the heart, there are several limitations. First, our study relied on public databases, yet each database has a different focus, and the lack of timely updating of the databases also led to the incompleteness of the data. Second, the components of TCM compounds are complex, and there is a discrepancy between in vitro theoretical studies and in vivo metabolism, so the results of data analysis only indicate the prediction results. Furthermore, we only investigated the potential genes of the effective compounds, and have not yet considered the toxicological effects of the compounds, as well as the synergistic and antagonistic effects among the compounds. considered the toxicological effects of the compounds as well as the synergistic and antagonistic effects among the compounds. Therefore, it is necessary for us to confirm the therapeutic effects of the drugs with a large amount of clinical data and to verify the exact molecular mechanisms through further animal experiments.

## 5. Conclusions

In this research, the results of network pharmacology and molecular docking techniques indicate that SGC may exert antiinflammatory effects by modulating IL-1β to improve cardiac dysfunction and thus treat HF.

## Author contributions

Visualization: Xiang Luo.

Writing—original draft: Xiang Luo.

Writing—review & editing: Xiang Luo, Zhao Hu.

Data curation: Yunke Shi, Yiming Ma, Yixi Liu, Pan Jing, Xingyu Cao, Jincheng Wang.

Formal analysis: Yunke Shi, Yiming Ma, Yixi Liu, Pan Jing, Xingyu Cao, Jincheng Wang.

Conceptualization: Zhao Hu, Hongyan Cai.

Funding acquisition: Zhao Hu, Hongyan Cai.

Supervision: Zhao Hu, Hongyan Cai.
